# High Hydrostatic Pressure: Influences on Allergenicity, Bioactivities, and Structural and Functional Properties of Proteins from Diverse Food Sources

**DOI:** 10.3390/foods13060922

**Published:** 2024-03-18

**Authors:** Sukan Braspaiboon, Thunnop Laokuldilok

**Affiliations:** Faculty of Agro-Industry, Chiang Mai University, Chiang Mai 50100, Thailand; sukan.bras@cmu.ac.th

**Keywords:** high pressure processing, unfolding, surface hydrophobicity, texture, allergenicity

## Abstract

High hydrostatic pressure (HHP) has gained prominence in the food processing industry over the last decade. In addition to the effectiveness of microbial and enzymatic inactivation, HHP directly impacts protein structures and properties. Accordingly, this review article aims to consolidate relevant research findings elucidating the effects of HHP on protein structure, allergenicity, bioactivities, and functional properties across diverse protein sources. They encompass cereals, legumes, nuts, meat, poultry products, milk, eggs, seafood, algae, insects, seeds, and vegetables. This review provides insights into the consistent trends of HHP effects on each protein source. In conclusion, HHP induces alterations in non-covalent bonds within protein structures, leading to the unfolding of their interior regions and consequential changes in their properties. Remarkably, the allergenicity of cereals, legumes, and nuts decreases while their bioactivities and digestibility escalate. The disruption of non-covalent bonds during HHP results in the exposure of the interior hydrophobic regions to the surface microenvironment, thereby enhancing the surface hydrophobicity of proteins, particularly those derived from seeds and vegetables. HHP weakens the allergenicity and elevates the foaming properties of proteins from dairy products, including improving the gelling properties and antioxidant activities of egg proteins. Texture profiles of meat and poultry, particularly hardness, are enhanced. Furthermore, HHP demonstrates the potential to diminish the allergenicity of seafood proteins and augment insect protein bioactivities. Lastly, HHP enhances the extraction of algal bioactive components, improving their nutritional quality.

## 1. Introduction

Proteins, which are essential nutrients for organisms, are derived from various sources, including animals (eggs, milk, meat, poultry, seafood, and insects), plants (cereals, legumes, nuts, seeds, and vegetables), and algae. Their applications are multifaceted, with each protein source exhibiting distinct amino acid profiles influencing various functional properties. Structurally, proteins adopt specific conformations determined by varied amino acid compositions and molecular interactions, including disulfide bridges, electrostatic interactions, hydrogen bonds, and hydrophobic forces [1]. Consequently, proteins exhibit diverse functional properties, such as gelation, solubility, surface hydrophobicity, and water-holding capacities. The inherent amphiphilic nature of proteins, characterized by hydrophobic and hydrophilic regions, makes them valuable in food additives, serving as emulsifiers and foaming agents [2].

Proteins provide nutraceutical properties, including antioxidant, angiotensin-converting enzyme (ACE)-inhibitory activities, and anti-inflammatory effects [3]. Despite these benefits, proteins are recognized as significant contributors to food allergens, triggering immune responses. Moreover, certain protein sources, particularly plants, encounter challenges in nutritional value due to the presence of antinutritional factors. These components directly hinder the digestibility and bioavailability of proteins [4]. Consequently, food processing plays a crucial role in modifying protein properties on various fronts, enhancing not only functional properties but also nutritional qualities to meet application or consumer requirements.

In terms of production processes, proteins are sensitive to various environmental factors such as pH, temperature, and pressure. Thermal processing, widely adopted in food industries, has traditionally been utilized for cooking and disinfection of contaminated microorganisms. While effective in pasteurization and extending the shelf life of food products, the heating process concurrently alters their structures and nutritional properties. For example, it can expedite the Maillard reaction, thereby impacting the development of caramel aroma in soy sauce [5,6]. Consequently, many food manufacturers have sought alternative processes to preserve nutritional components and textures in line with consumer preferences. In the current decade, numerous emerging nonthermal technologies have been researched and introduced in the food industry, driven by concerns about carbon dioxide emissions and global climate conservation. High-pressure processing or high hydrostatic pressure (HHP) stands out as a non-thermal technology employed for decontamination in the food industry. Recognized for its eco-friendly approach, HHP has gained attention [7]. It has been extensively employed for commercial pasteurization, causing cell destruction while preserving bioactive substances in food components. However, proteins, shaped by intra- and intermolecular interactions, may be influenced by this process. Recent research has delved into the effects of HHP on the structure and functional properties of various protein sources subjected to this technology. Studies have reported alterations in structures, functional properties, and bioactivities, including allergenicity. Despite this, there remains a gap in reviews that compile the results of comprehensive protein sources affected by HHP. To address this gap, this review aims to aggregate recent articles (2014–2024) in reliable online academic publishers that investigate the impact of HHP on the structural and functional properties, bioactivities, allergenicity, and other relevant attributes of various dietary proteins. This review provides insights into trends and overall changes resulting from the HHP application.

## 2. Principle of HHP and Its Role in Food Processing

**Figure 1:**
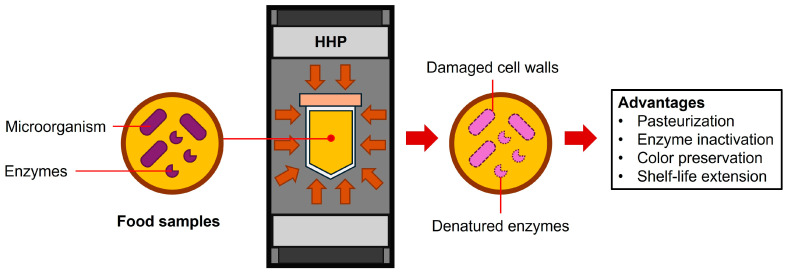
The application of HHP technology in food industries.

The principle underlying HHP execution relies on pressurization through volumetric reduction, aligning with Le Chatelier’s principle. In this process, food samples from diverse protein sources are first suspended in solvents, and then they are enclosed in sealed packaging before being placed into a pressure chamber containing a fluid medium (Figure 1). The volumes within the pressure chamber are subsequently reduced by the compression of a hydraulic pump [8]. This reduction in volume leads to sudden pressurization in the medium. Under HHP, this pressure is transmitted uniformly throughout the fluid medium. Concurrently, the pressure acts uniformly on the food surface, enclosing it in all directions with the same magnitude, following the isostatic principle of Pascal’s law [9]. Eventually, food materials respond to exterior pressure in the opposite direction, influencing the alteration of their component structure [8].

In recent years, HHP has gained widespread adoption in the food industry, primarily aiming to replace thermal processing with pasteurization methods. While thermal processes effectively reduce microbial contamination, they may also compromise bioactive components sensitive to heat. HHP, based on structural alterations during pressurization, induces damage to microbial cell structures without the need for thermal input, thus preserving the nutritional values of food products [10]. HHP has been extensively employed in the pasteurization of various foods, including dairy products, juice, meat, and seafood. Pressurization at 600 MPa has demonstrated a remarkable reduction (5–8 logs) in a broad spectrum of microorganisms, such as *Escherichia coli*, *Salmonella enteritidis*, *Listeria monocytogenes*, and *Vibrio* sp. [11]. Beyond microbial pasteurization, HHP has been utilized to suppress enzymatic activity, preventing browning in juice products with pressure applied at 400–600 MPa for five minutes [12]. This application not only preserves vitamins in juice but also retains the product’s colors, influencing consumer purchasing decisions.

## 3. Effects of HHP on Allergenicity, Bioactivities, as well as Structural, Functional, and Other Properties of Proteins

Proteins represent a diverse class of bio-macromolecules, composed of twenty different types of amino acids. Each amino acid consists of a carboxyl group (COOH) and an amino group (NH_2_), with variations occurring in their respective side chains (R group). The formation of peptide bonds involves electron sharing between the carboxyl group and the amino group of adjacent amino acids, constituting a covalent bond. The long chains of amino acid sequences linked with many peptide bonds are called polypeptide chains, which are primary structures. In addition to covalent bonds, polypeptide chains can form intramolecular interactions through numerous non-covalent bonds, such as hydrogen bonding, hydrophobic interactions, and electrostatic interactions, including van der Waals forces. These non-covalent interactions cause different protein conformations. For example, intramolecular hydrogen bonds, formed by a carbonyl group (C=O) and hydrogen atom of another amino group, generate different shapes of secondary structures, such as alpha-helix (α-helix), beta-sheet (β-sheet), and random coils. These structures can be included within the same polypeptide chain. Moreover, different amino acids have various side chains with both hydrophilic and hydrophobic properties. The side chains can form intramolecular interactions, such as disulfide bonds from sulfur amino acids, ionic interactions between different amino acid charges, and hydrophobic interactions within the same polypeptide backbones [13].

**Figure 2:**
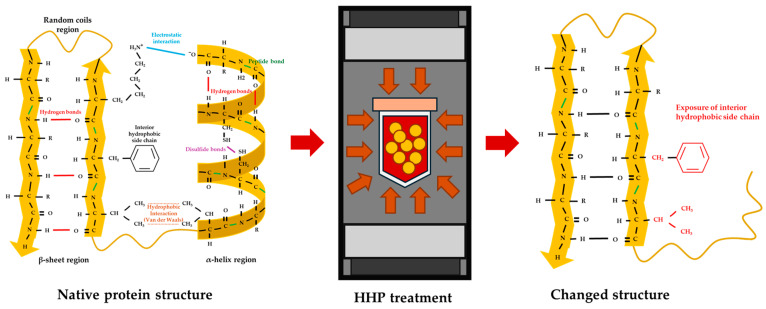
The simulated image of secondary structure alteration posterior HHP [13,14,15].

According to these diverse interactions and amino acid sequences, the three-dimensional structures, combined with multiple polypeptide subunits, exhibit specific native conformations and distinct properties, including allergenicity, bioactivities, and functional properties. Non-covalent bonds, being inherently weak, can be disrupted and reformed based on external factors such as ionic strength, pH, temperature, and pressure [14]. The disruption of non-covalent bonds directly impacts the contents of α-helix and β-sheet (Figure 2), consequently influencing the mentioned protein properties. For instance, the heating process of flaxseeds led to an increase in β-sheet and a decrease in α-helix due to intermolecular β-sheet formation. This formation influenced the functional properties of flaxseed proteins by inducing protein aggregation and reducing solubility. Moreover, this transformation probably involved the alteration of nutritional values. The elevated β-sheet formation from protein aggregation hindered enzyme accessibility, thereby influencing the reduction in protein digestibility [15].

In general, HHP has been applied in pasteurization processes as an alternative to thermal methods to preserve nutritional value for consumers. Meanwhile, HHP can modify the molecular structures of some food ingredients, such as the non-covalent bonds of starch [16]. Likewise, the modification of non-covalent bonds directly affects protein properties [14]. Recently, HHP has been utilized to modify various protein properties, with a predominant focus on functional attributes. Numerous studies have consistently reported alterations in hydrophobic, emulsifying, and foaming properties under HHP conditions. Texture profiles, including hardness, springiness, chewiness, and gumminess, have also been frequently investigated. HHP exhibits the potential to influence enzymatic activities, thereby affecting protein digestibility, antioxidant activities, and ACE inhibitory activity. Notably, HHP fundamentally influences the allergenicity of proteins from various sources, a critical aspect of ensuring consumer safety. This review provides an in-depth exploration of the HHP impact on these properties for each protein source.

### 3.1. Plant-Based Protein Sources

#### 3.1.1. Cereals

Cereals, as primary crops crucial for the global population, serve as a prominent protein source, second only to legumes. However, they are also recognized as significant allergenic sources, exemplified by gluten from wheat, which can cause celiac disease. Consequently, many individuals abstain from consuming proteins derived from these sources. In addition to enzymatic hydrolysis, HHP has been recognized for its capacity to reduce allergens in cereal proteins (Table 1). Specifically, HHP has been reported to diminish the allergenicity of buckwheat, with a reduction of 72.2% achieved at 400 MPa for 20 min [17]. This effect is attributed to HHP-induced destruction or aggregation of proteins, leading to a decrease in epitopes and subsequently reducing immunoglobulin E binding capacity [17]. Furthermore, this processing methodology has been observed to enhance antioxidant activities in proteins from buckwheat [18] and rice [19,20] when subjected to high pressure at 600 and 300–400 MPa for 15–30 min, respectively. Despite limited explanations regarding the HHP effect on antioxidant activities, it is noteworthy that the antioxidant activities of rice protein hydrolysate treated by HHP surpassed those of both rice protein isolates and hydrolysates. This outcome can be elucidated by HHP-induced disassociation of protein structures, facilitating enzyme activities [20], and is correlated with a smaller molecular distribution. HHP generates lower molecular weight peptides, offering potential advantages for antioxidant activities and protein digestibility [20].

Regarding functional properties, the surface hydrophobicity of proteins subjected to HHP exhibited variations (Table 1). Millet protein [23] and quinoa protein [27] demonstrated a decrease in this property after being treated at 500–600 MPa for 10–15 min, while rice proteins [19,24] and wheat protein [17] exhibited an increase at >300 MPa. This disparity was found to be associated with changes in fluorescence intensity. High pressure induced the folding of the hydrophobic region in gliadin, resulting in a reduction in fluorescence intensity [23]. Conversely, HHP disrupted hydrophobic interactions, exposing the hydrophobic groups of rice protein to the surface, leading to an increase in fluorescence intensity [25]. Additionally, the reduced hydrophobicity resulted from protein aggregation, with HHP exposing buried tryptophan residues to the microenvironment, thereby triggering aggregation. Protein aggregation was further evidenced by an increase in particle size and a reduction in zeta potential in millet protein [23] and quinoa protein [27], impacting structural forms. The decrease in α-helix content converted to β-sheets contributed to a decline in surface charge and electrostatic repulsion, further promoting protein aggregation [23]. However, variations in α-helix and β-sheet contents across protein samples were probably caused by diverse amino acid sequences within each protein sample. Accordingly, HHP probably resulted in different contents of changes in secondary structures. In addition, indices of emulsifying activity (EAI) and stability (ESI), as well as foaming capacity tendencies, were found to correspond to hydrophobicity. The increase in these properties in rice protein [19,24] and rice bran protein [25] was associated with higher hydrophobicity, contrasting with the behavior observed in buckwheat protein [18]. In terms of texture, HHP demonstrated the capability to enhance storage modulus and viscosity in proteins derived from barley [22], quinoa [26], and wheat [29]. The increased storage modulus and gel strength were attributed to hydrophobic interactions following protein unfolding under HHP [29].

#### 3.1.2. Legumes and Nuts

Legumes have emerged as the primary source of plant proteins, boasting an overall protein content that surpasses that of other botanical groups. Presently, global priorities in nutrition and environmental impacts have led to the substitution of animal-based proteins with these plant-derived protein sources, particularly in industries like supplements and food hydrocolloids. In the realm of food supplements, soy proteins have gained prominence due to their amino acid composition closely resembling that of animal-based proteins, notably whey protein. However, significant drawbacks persist in terms of allergenicity and protein digestibility for soy-based products. Thus, HHP has been employed to enhance their nutritional properties as shown in Table 1. HHP has proven effective in mitigating the allergenicity of legumes, notably soybeans [43,45,46], peanuts [40], and almonds [30]. The increase in pressure was associated with a concurrent reduction in allergenicity. Furthermore, HHP treatment has been demonstrated to improve the protein digestibility of soy protein [42] and walnut protein [41]. In the case of soybeans, agglutinin has emerged as a primary anti-nutritional factor inhibiting gastrointestinal enzymatic activity, and high-pressure application was found to diminish the activity of soybean agglutinin [42]. Moreover, the antioxidant and ACE inhibitory activities of legume proteins, such as those from soybeans, exhibited an increase with pressure elevation from 80 to 300 MPa [44].

In the context of functional properties, emulsion properties have been a focal point in discussions due to the impact of HHP. Indices such as EAI and ESI in various legumes, including kidney beans [36], peas [37], soybeans [47,48], walnuts [41], lentils, and faba beans [38], were notably influenced by HHP (Table 1). An increase in EAI occurred due to protein unfolding under HHP, leading to an augmented hydrophobic exposure [47]. Additionally, ESI was impacted by droplet size, decreasing under higher pressure. The heightened hydrophobic regions exposed under increased pressure facilitated interaction with oil droplets, resulting in smaller emulsified particles [37]. Similarly, the increased hydrophobic sites revealed by higher pressure promoted greater surface hydrophobicity in legume proteins from peanuts [39], soybeans [47,48,49], and walnuts [41]. This increment in surface hydrophobicity further enhanced the foaming capacity and stability of kidney beans [36], soybeans [48], walnuts [41], and pulse proteins [38]. Moreover, changes in conformation, such as a reduction in β-sheet content, were associated with improved emulsifying and foaming properties. Yan et al. [48] reported that decreased β-sheet content can enhance these properties of soy protein isolates. These improved properties support the application of proteins modified by HHP. For example, the use of an emulsifier derived from soy protein isolate treated by HHP at 250 MPa for 30 min can enhance the melting rate and reduce the hardness of ice cream, leading to better product acceptability. Conversely, the solubility of proteins treated by HHP displayed inconsistency. The solubility of proteins from almonds [30], cowpeas [32], peas, lentils, and faba beans [38] decreased, while that of proteins from soybeans [47,48,49] and walnuts [41] increased. These different outcomes were likely a result of the degree of denaturation. The appropriate pressure range (200–400 MPa) induced partial protein unfolding without complete denaturation, thereby enhancing protein solubility for soy protein isolates treated under this pressure range. However, further pressure exceeding 400 MPa reduced solubility due to the formation of new disulfide bonds, limiting protein unfolding and causing lower solubility [33]. Concerning the effect on water holding capacity, the trend was unidirectional with pressure magnitude in proteins from cowpeas [31], kidney beans [36], peanuts [39], peas, lentils, and faba beans [38]. The increase in water holding capacity resulted from the red-shift phenomenon observed in fluorescence spectroscopy. The reduction in fluorescent intensity was a consequence of protein unfolding, revealing additional polar regions of tryptophan residues [31].

#### 3.1.3. Seeds

Seeds, consumed by humans for their palatability, are also recognized as valuable sources for plant oil industries, with by-products containing relatively high protein content. Consequently, they have been employed in the production of seed proteins for various food applications. In recent times, HHP has been employed to enhance the functional properties of different seed proteins, as outlined in Table 1. HHP has been consistently reported to augment seed protein hydrophobicity and antioxidant activity. For instance, Zhou et al. [53] investigated the impact of HHP on the modification of ginkgo seed proteins using pressures ranging from 100 to 700 MPa for a duration of 20 min. They observed a reduction in the structural proportions of α-helix and β-sheet structures, accompanied by an increase in random coils. A critical pressure of 400 MPa was identified for the disruption of α-helix and β-sheet structures, leading to their transformation into a random coil. Similarly, structural modifications occurred under high-pressure treatment at 400 MPa, where a treatment duration of 15 min resulted in the maximum hydrophobicity in rapeseed protein [54]. This observed phenomenon aligned with findings in cumin seed protein, treated at 600 MPa for 5 min, displaying a comparable pattern of structural change. This alteration was induced by the interaction between secondary and tertiary structures, as evidenced by the elevated level of sulfhydryl content. The increased sulfhydryl content signifies the exposure of the protein and subsequent backbone fragmentation. Furthermore, HHP also influences fluorescence intensity and UV absorption, supporting the phenomena of protein unfolding. The decreased fluorescence intensity reflected higher surface hydrophobicity, resulting from the exposure of hydrophobic regions to the microenvironment, represented by tyrosine and tryptophan residues. Furthermore, emulsifying activities, including EAI and ESI, were influenced by the heightened surface hydrophobicity induced by increased pressure. The elevated pressure exposed hydrophobic residues to the environment, leading to a higher EAI value in ginkgo seed proteins [53]. However, the impact of HHP on cumin seed proteins differed. The values of EAI and ESI decreased with higher pressure, attributed to protein aggregation and the burial of surface hydrophobic regions, resulting in lower EAI and ESI [50]. Under high HHP, protein aggregation increased due to the formation of disulfide bonds, leading to improved gelation properties in cumin seed protein, as evidenced by a higher storage modulus [50].

Moreover, the alteration in protein structure under HHP provides an additional benefit to consumers by reducing allergenicity (Table 1). The allergenicity of ginkgo seed proteins decreased with increasing pressure, with samples treated above 600 MPa showing an absence of immunoglobulin E binding activities [53]. Additionally, HHP demonstrated the capacity to enhance the antioxidant activities of flaxseed protein samples, as assessed by the oxygen radical absorbance capacity (ORAC) assay after hydrolysis by either trypsin [52] or tryptic-pronase [51]. The disassociation of protein interactions induced by HHP led to protein unfolding, revealing structures conducive to enzymatic activities, and resulting in higher antioxidant activities in seed proteins [51,52].

#### 3.1.4. Vegetables

Vegetables are generally not considered significant protein sources due to their primary composition being carbohydrates. However, various research studies have investigated root vegetables, such as sweet potatoes, utilizing wastewater from starch production processes to derive proteins for emulsifiers [56]. Sweet potatoes contain notable proteins, namely Sporamin and Patatin [60], contributing functional properties and nutritional value. HHP has been employed to modify the functional properties of sweet potato proteins for commercial applications, as presented in Table 1. The observed tendency of HHP effects, involving pressures ranging from 200 to 600 MPa and treatment durations between 15 and 30 min, revealed a consistent pattern on sweet potato proteins. HHP induced the disruption of both intramolecular and intermolecular interactions within sweet potato proteins by decreasing the α-helix conformation while increasing the proportion of β-sheet. The changes in conformation were linked to protein interactions. The stability of the α-helix conformation depended on intramolecular hydrogen bonding, whereas the β-sheet was stabilized by stronger intermolecular hydrogen bonding. Consequently, the proportion of α-helix decreased after HHP treatment [61].

The disruption of bonding also exerted a profound impact on the functional properties of sweet potato proteins (Table 1). Primarily, several studies consistently reported an increase in surface hydrophobicity [58,60,62,67]. The peak surface hydrophobicity was achieved through the unveiling of the sweet potato protein structure around 400–450 MPa, exposing the interior hydrophobic region to the external environment [60]. Additionally, protein unfolding, indicative of protein denaturation, altered thermal enthalpy. The change in enthalpy (∆H), representing the fraction of non-denatured protein, decreased, signifying a higher degree of protein denaturation [59]. Furthermore, HHP augmented the sulfhydryl content of sweet potato proteins, influencing its rheological properties. The higher sulfhydryl content encouraged cross-linking interactions with disulfide bonds between proteins, inducing protein aggregation [61]. Consequently, the storage modulus of sweet potato proteins increased, and texture profiles, including gel hardness, springiness, chewiness, and gumminess, as well as viscosity, were improved [63]. In emulsion systems, the exposure of hydrophobic sites following HHP treatment decreased surface tension, thereby enhancing EAI and ESI values [58]. Regarding bioactive activity, the unfolding of the protein structure after treatment in the range of 450–550 MPa also promoted the bioactivities of sweet potato proteins, assessed through DPPH radical scavenging activity, ORAC, and ferrous ion-chelating activity [60,67], including ACE inhibitory activity [65]. The enhanced bioactive activities resulted from the unwinding of the protein structure under HHP, fostering enzymatic activities and bioactive properties of protein hydrolysates [67].

### 3.2. Animal-Based Protein Sources

#### 3.2.1. Dairy Products and Eggs

Milk and eggs represent fundamental protein sources, providing nutritional value to meet body requirements through their relatively high amounts of essential amino acids and demonstrating high protein digestibility. Consequently, these sources, particularly eggs, have become pivotal benchmarks for evaluating protein quality. In the past decade, protein supplements have gained prominence in modern diets due to changing dietary behaviors, with functional foods like whey proteins becoming popular choices. However, whey proteins can induce allergies in some individuals due to the presence of beta-lactoglobulin. Consequently, HHP has been employed to isolate alpha-lactalbumin from beta-lactoglobulin [68], as shown in Table 2. The application of HHP has resulted in a reduction in beta-lactoglobulin content in whey proteins [68,69] and whole milk [70]. The allergenic reduction in dairy products is attributed to decreased beta-lactoglobulin content, achieved through HHP treatment at 600 MPa for at least 5 min [69,70]. The structural rigidity of alpha-lactalbumin surpasses that of beta-lactoglobulin due to the presence of a higher number of disulfide bonds. While the tertiary structure of alpha-lactalbumin is established by 4-disulfide bonds, beta-lactoglobulin possesses only 2-disulfide bonds. Consequently, beta-lactoglobulin is more sensitive to HHP treatment than alpha-lactalbumin [68]. Furthermore, HHP treatment has been shown to diminish the allergenicity of whey proteins [71,72], including the reduction of secretory immunoglobulin A levels in skim milk [73]. The decrease in allergenicity is attributed to the disruption of protein conformation under HHP. The exposure of hydrophobic groups after HHP treatment induces intermolecular interactions between protein molecules, leading to the masking of antigenic epitopes [71]. This observation aligns with the increased surface hydrophobicity in whey proteins following HHP treatment [71,72,74].

The HHP treatment significantly enhanced the absorption capacity of air bubbles within whey proteins (Table 2), resulting in improved foaming properties [75]. The enhanced foaming properties can be attributed to the reduced interfacial tension, which stems from the unveiling of the protein structure under HHP. Pressures exceeding 300 MPa induced protein unfolding, leading to increased surface hydrophobicity. This unfolding phenomenon exposed hydrophobic amino acid residues to a polar environment of accessible solvents [72,74]. Consequently, HHP promotes surface hydrophobicity, foaming properties, and ESI. The heightened stability of the interfacial film in whey proteins, attributed to elevated storage modulus and micro-viscosity, resulted from the formation of intermolecular hydrophobic interactions induced by HHP [76]. In the context of egg proteins, limited literature addresses the impact of HHP. Specifically, subjecting liquid whole egg, egg white, and egg yolk to HHP at 900 MPa for 15 min results in heightened hardness. This increase can be attributed to pressure-induced gelation, arising from protein aggregation under HHP conditions, as reported by Singh and Ramaswamy [77].

Additionally, subjecting egg white protein to trypsin hydrolysis under HHP conditions (550 MPa for 15 min) led to an increase in both the degree of hydrolysis and antioxidant activity, as determined by the DPPH radical-scavenging assay (Table 2). The dissociation of the white egg protein structure induced by HHP was found to enhance the functionality of trypsin activity [78]. Antioxidant activities have also elevated the application of HHP in whey proteins [72,79,80] and caseins [81]. The increased antioxidant activities of dairy products were validated using an oxidative damage cell model in human intestinal epithelial cells (Caco-2 cells. When Caco-2 cells were treated with whey protein under HHP, there were lower levels of reactive oxidative species and malondialdehyde. Additionally, the expression of antioxidant enzymes like superoxide dismutase, catalase, and glutathione peroxidase increased, reaching its peak at 600 MPa [75]. Furthermore, HHP elevated the protein digestibility of whey proteins [71,79,80] caseins [81]. Protein digestibility correlated with a higher degree of hydrolysis during HHP in whey proteins [75,82]. 

**Table 2:** The effects of HHP on bioactivities, allergenicity, as well as structural, functional, and other properties of animal-based protein sources.

Protein Sources	Samples	Extraction Methods	Pressure (MPa)	Time (min)	Effects on Samples			References
					Structural Property	Bioactivities and Allergenicity	Functional and Other Properties	
Dairy products and eggs	Whey protein isolates	-	550	1	-	(+) antioxidant activity	(+) protein digestibility	[79]
		-	100–400	5–30	(+) SH	-	(+) DH	[82]
		-	600	10–15	(−) β-Lg (+) FI	-	(+) turbidity (+) protein yield (+) purification degree	[69]
		-	100–600	15–30	(+) α-helix, β-sheet (+) SH	-	(+) DH (+) foaming capacity (+) foaming stability (−) interfacial tension	[75]
		-	600	10	(+) α-helix (−) β-sheet (+) FI	-	(+) G′ (+) surface hydrophobicity (−) interfacial tension (+) micro-viscosity	[74,76]
		-	500	5–15	(+) α-helix (+) FI	(−) allergenicity	(+) surface hydrophobicity (+) protein digestibility	[71]
		-	100–600	30	(−) α-helix, β-sheet (+) random coil (+) FI (+) SH	(+) antioxidant activity	(+) DH (+) protein digestibility	[80]
		-	100–600	10–20	(+) α-helix, β-turn (−) β-sheet (+) FI (+) SH	(−) allergenicity (+) antioxidant activity	(−) EAI (+) surface hydrophobicity	[72]
	Casein extracts	Acid (pH 4.6)	200–600	5–15	-	(+) ACE-inhibitory activity (+) antioxidant	(+) protein digestibility	[81]
	α-La and β-Lg milk powders	-	200–600	1.7–5	(−) β-Lg	-	(+) protein recovery (+) purification degree of α-La	[68]
	Whole milk	-	600	5	(−) β-Lg	-	-	[70]
	Skim milk	-	250–900	5	(−) particle size	(−) secretory IgA	(−) protein solubility	[73]
	Eggs	-	600–900	5–15	-	-	(+) hardness	[77]
	Egg white	-	350–550	5–15	-	(+) antioxidant activity	(+) DH	[78]
Meat and poultry products	Bovine serum albumin	-	100–600	15	(+) SH	-	(+) foaming capacity (+) EAI and ESI	[83,84]
	Rabbit myosin extracts	KCl (0.6 M)	100–200	2	(−) α-helix (+) β-sheet, β-turn (−) droplet size	-	(+) interfacial tension (+) EAI and ESI (+) G′	[85]
	Beef jerky	-	100–300	20	(+) TBARS	-	(−) tenderness (−) moisture content (+) L* and (−) a*	[86]
	Beef gel	-	100–200	10	-	-	(−) L*, a*, and b* (+) G′ (+) hardness and adhesiveness	[87]
	Beef patties	-	300	5	-	-	(+) cooking loss (−) expressible moisture (+) L* and (−) a*, b* (−) G′ (+) hardness, springiness, and chewiness (+) water release	[88]
	Beef rounds	-	300–600	5	(+) C=O	-	(+) L*, b* and (−) a*	[89]
	Chicken meat	-	50–200	1–3	-	-	(+) L* (−) expressible moisture (+) hardness, cohesiveness, and chewiness	[90]
	Chorizos	-	600	8	(+) C=O (+) TBARS	-	-	[91]
	Frankfurters	-	300–600	4	-	-	(+) firmness (+) drip loss	[92]
	Lamb cuts	-	200–600	1	(+) TBARS	-	(+) free AA	[93]
	Wieners	-	600	3	(+) TBARS	-	(−) expressible moisture (−) hardness, springiness, and chewiness	[94]
Seafood	Bighead carp protein extracts	KCl (0.6 M)	300	20	(−) α-helix, random coil (+) β-sheet (−) FI (−) SH	(+) antioxidant activity	(+) DH (+) surface hydrophobicity (+) zeta potential	[95]
	Cod protein extracts	Tris-maleate (0.02 M, pH 7)	200	20	-	(+) ACE inhibitory activity (+) anti-inflammatory activity (+) antioxidant activity	(+) AA content	[96]
	Eel (surimi) Protein extracts	PBS (0.1 M, pH 7.5)	100–600	5	(+) β-turn, random coil (+) SH	(+) ACE inhibitory activity	(+) surface hydrophobicity (+) hardness, adhesiveness, and chewiness (+) WHC	[97]
	Oyster protein extracts	PBS (0.05 M, pH 7)	300–600	5	(−) α-helix (+) β-turn, β-sheet, random coil (−) FI (+) SH	(−) IgG binding capacity	(+) surface hydrophobicity	[98]
	Scallop protein extracts	PBS (0.05 M, pH 7)	100–500	10	(−) α-helix (+) β-sheet (+) SH (+) droplet size	-	(+) surface hydrophobicity (+) zeta potential (+) ESI and EAI (+) creaming index (+) protein adsorption (−) protein solubility	[99]
	Squid protein extracts	Protein lysis buffer	200–600	20	(−) α-helix (+) β-sheet, random coil	(−) allergenicity	(+) surface hydrophobicity (+) protein digestibility	[100]
	Alaska pollock (surimi)	-	300	10	(−) ∆H	-	(+) WBC (+) breaking force	[101]
	Large yellow croaker	-	300–600	10	(−) α-helix (+) β-sheet, random coil (−) FI (−) SH	(−) allergenicity	(+) surface hydrophobicity	[102]
	Red abalone	-	200–500	-	(+) β-sheet	-	(+) protein digestibility	[103]
	Razor clam	-	200–400	1–10	(+) TBARS	(−) Ca^2+^-ATPase activity	(+) WHC (−) drip loss (+) pH (−) protein content	[104]
	Silver pomfret	-	100–200	-	(−) SH (+) C=O	(−) Ca^2+^-ATPase activity	(+) surface hydrophobicity (+) thawing loss (−) WHC (+) hardness, springiness, chewiness, and gumminess (+) L* and b* (+) pH	[105]
	Threadfin bream	-	200–600	10–50	(−) SH	(−) Ca^2+^-ATPase activity	(−) protein solubility (+) surface hydrophobicity (+) turbidity	[106]
	Tilapia (surimi)	-	100–400	15	-	-	(+) L* (+) WHC (+) gel strength (+) hardness, springiness, and chewiness	[107]
	Oysters	-	100–500	5	-	-	(+) hardness, springiness, chewiness, and cohesiveness	[108]
	Shrimps	-	550	5	(−) TBARS	-	(+) moisture content (+) G’ (+) L* (−) a* and b* (−) firmness	[109]

Symbols: (−) decrease; (+) increase. Abbreviations: AA (amino acid); angiotensin-converting enzyme (ACE); C=O (carbonyl); DH (degree of hydrolysis); EAI (emulsifying activity index); ESI (emulsifying stability index); FI (fluorescence intensity); G′ (storage modulus), IgA (immunoglobulin A); IgG (immunoglobulin G); KCl (potassium chloride); L* (lightness); a* (red/green values); b* (blue/yellow values); PBS (phosphate buffer saline); SH (sulfhydryl); thiobarbituric acid reactive substances (TBARS); WBC (water binding capacity); WHC (water holding capacity); ∆H (enthalpy change); α-La (alpha-lactalbumin); β-lactoglobulin (β-Lg).

These outcomes were a result of protein unfolding after HHP, facilitating higher enzymatic activities, increased degree of hydrolysis, and enhanced protein digestibility [69]. HHP applied at 400 MPa for 30 min improved the degree of hydrolysis, with the degree of hydrolysis value positively correlating with pressure magnitude and treatment time. The unraveling of the tertiary structure of whey proteins by HHP promoted enzyme activities, resulting in a higher degree of hydrolysis and improved protein digestibility [69].

#### 3.2.2. Meat and Poultry Products

Meat and poultry products have long been primary protein sources in various human dishes, where their texture and color play pivotal roles in consumer satisfaction. Traditional cooking methods, such as heating, are employed for decontamination, leading to protein denaturation. However, excessive heating can result in the loss of desired functional properties and a reduction in nutritional value due to nutrient destruction. To address these challenges, HHP has been utilized to preserve the qualities of meat and poultry products for consumers [92], ultimately enhancing consumer satisfaction. Color is a crucial property significantly influenced by the application of HHP. The method affects the discoloration of meat and poultry products by increasing lightness and reducing redness under high pressure in various samples, including ground beef [89], chicken meats [90], beef gel [87], beef patties [88], and beef jerky [86], (Table 2). These observed changes can be attributed to protein denaturation, particularly of myoglobin, and the release of heme, contributing to increased lightness under high pressure [89]. Additionally, HHP reduces redness by mitigating the oxidation of ferrous myoglobin to ferric brown metmyoglobin [87]. It is noteworthy that the color shift toward blue or yellow remains unaffected by this treatment [90].

Texture is a pivotal aspect significantly influenced by HHP treatment. Numerous studies have consistently reported that HHP can enhance the texture profiles of products, including hardness, springiness, cohesiveness, chewiness, and adhesiveness, (Table 2). The improvement in texture results from the stronger interactions between proteins that are regenerated under high pressure. These enhanced protein interactions contribute to a homogeneous gel microstructure, ultimately increasing the hardness of the texture [87]. However, HHP can also have negative impacts on certain product qualities. For instance, there is an observed increase in cooking loss or drip loss in products such as beef patties [88] and frankfurters [92], leading to lower moisture content in chicken meat [90] and beef jerky [86]. This increased cooking loss is accompanied by lower water binding capacity properties, a result of denatured proteins induced by HHP, negatively affecting the water retention of the products [88]. Moreover, HHP has been linked to elevated lipid oxidation in various meat products, including lamb cuts [93], Chorizos sausage [91], and beef jerky [86], as indicated by measurements of thiobarbituric acid reactive substances. This could potentially impact consumer acceptance of meat and poultry products. The heightened lipid oxidation is attributed to the release of metal ions, which promote free-radical catalyzed oxidation [86]. In addition to these effects, HHP has been reported to enhance the activity and stability of emulsions in samples of rabbit muscle proteins [85] and bovine serum albumin [84].

#### 3.2.3. Seafood

Seafood, while constituting a protein source globally consumed, does not enjoy the same prominence as meat and poultry, primarily due to its elevated cost and the prevalence of allergies to seafood proteins among individuals, posing a significant drawback for this protein source. Recently, HHP has emerged as a potential solution to mitigate allergenicity in various types of seafood (Table 2). Notably, the allergenicity of tropomyosin Tod p1, a major allergen in squid, exhibited a reduction when subjected to pressures exceeding 200 MPa for 20 min. The binding reactivities of immunoglobulin E and immunoglobulin G were notably low at 400 MPa, with no significant difference observed at 600 MPa [100]. Similarly, oyster tropomyosin demonstrated outcomes parallel to those of squid, with the lowest immunoglobulin G binding activity observed at 600 MPa for 5 min [108]. The diminished allergenicity was attributed to conformational changes induced by HHP. The unfolding of native proteins under HHP conditions altered their tertiary structure, subsequently hiding the active immunoglobulin G binding site within the structure and thereby reducing IgG binding capacity [108]. This phenomenon correlated with higher surface hydrophobicity observed under HHP treatment in various seafood, including threadfin bream [106], squid [100], silver pomfret [105], large yellow croaker [102], eel surimi [97], bighead carp [95], scallops [99], and oysters [98]. HHP treatment exposed hydrophobic regions previously buried inside the structure, replacing the native active sites [98]. The resulting higher hydrophobicity led to an increase in droplet size, emulsion indicators (EAI and ESI), the creaming index, and a decrease in fluorescence intensity [99].

Seafood is frequently consumed in a fresh state, with texture and color being pivotal factors influencing purchasing decisions. In addition to its ability to destroy microorganisms, HHP has been successful in improving texture attributes such as hardness, springiness, and chewiness in different seafood types, as outlined in Table 2. This includes silver pomfret [105], tilapia [107], eel surimi [97], and oysters [108]. The improvement in texture profiles can be attributed to protein unfolding and the subsequent formation of protein interactions under HHP. Gel networks established under high pressure, particularly above 100 MPa, induce the formation of hydrogen bonds, as evidenced by higher sulfhydryl content in eel surimi [97], oysters [98], and scallops [99]. Notably, the modifications induced by HHP result in superior textures compared to traditional thermal treatments. HHP promotes the formation of hydrogen-bonded gels, leading to flexible gel networks that enhance the springiness and chewiness of seafood proteins [107]. Furthermore, the denaturation of proteins under high pressure exposes interior hydrophobic groups, thereby enhancing protein–lipid interactions [85]. This phenomenon aligns with the observed higher surface hydrophobicity under HHP treatment in various seafood, including threadfin bream [106], squid [100], silver pomfret [105], large yellow croaker [102], bighead carp [95], scallops [99], and oysters [98]. Similarly, HHP has demonstrated positive effects on moisture content in shrimp [109], resulting in a higher water holding capacity in tilapia [107], surimi from eel [97], and Alaska pollock [101], as well as lower drip loss in razor clam [104].

Regarding color value, various seafood samples, including silver pomfret [105], tilapia [107], and shrimp [109], exhibited increased lightness following HHP treatment. This outcome is attributed to protein coagulation and the loss of active pigments, particularly astaxanthin in shrimp [109]. Moreover, HHP has been observed to enhance the bioactivities of seafood samples (Table 2). For instance, improvements in antioxidant activity were noted in bighead carp [95], along with ACE inhibitory activity and anti-inflammatory activity in cod [96]. HHP, by disrupting the tissue matrix and inducing protein unfolding, encourages enzymatic activities. The higher enzymatic activity resulting from HHP treatment contributes not only to high antioxidant activities but also to ACE inhibitory activities in protein hydrolysates [95,96]. Furthermore, HHP has been shown to improve protein digestibility in red abalone [103] and squid [100]. This improvement is also associated with the elevated enzymatic activities facilitated by HHP.

### 3.3. Alternative Protein Sources

#### 3.3.1. Algae

Beyond animals and plants, algae have played a significant role in human diets since ancient times, falling into two main categories: macroalgae, commonly known as seaweed, and microalgae, notably cyanobacteria. Microalgae, such as *Arthrospira platensis* (spirulina), are recognized for their high protein content, making them valuable protein sources. Algae offer substantial competitive advantages in commercialization, requiring less arable land, emitting carbon-neutral emissions, and exhibiting a higher growth rate [110]. Consequently, algal proteins have emerged as a noteworthy contemporary protein source. Additionally, some algae, especially Rhodophyceae and cyanobacteria, are significant sources of essential bioactive compounds known as phycobiliproteins, which function as light-harvesting complexes providing diverse health benefits and applications. Phycobiliproteins can be classified into three groups: phycoerythrin, phycocyanin, and allophycocyanin, each contributing different spectral colors of pigments [111]. Moreover, prominent protein sources among algae include *Arthrospira platensis* and *Chlorella vulgaris*, both single-cell proteins with relatively high protein content. Consequently, they are commonly utilized as sources for protein extraction, including phycobiliproteins. The purity of extracted proteins and phycobiliproteins holds significance for various application objectives. Accordingly, HHP has been employed to enhance this extraction process (Table 3). HHP conditions ranging from 100 to 600 MPa within a 20-min duration were investigated for their impact on extracting phycocyanin content from *A*. *platensis* [112,113]. The results indicated that HHP can enhance total soluble protein, phycocyanin content, and purity. In terms of functional properties, HHP demonstrated the ability to elevate the storage modulus of biomass suspensions from both *A*. *platensis* and *C*. *vulgaris*, thereby influencing higher viscosity [114]. Furthermore, phytochemical extraction conducted at 400 MPa for 20 min was found to increase protein content, total phenolic content, and antioxidant activities of extracts [115]. Hence, HHP presents itself as a potential technique for extracting photosynthetic proteins from algae biomass.

#### 3.3.2. Insects

In recent years, there has been a growing interest in exploring alternative protein sources, with insect proteins emerging as a promising option due to their abundance and potential to meet global dietary demands. The application of HHP in the production processes of insect proteins, particularly focusing on enhancing enzymatic activity during hydrolysis, has gained attention (Table 3). Mealworms and crickets are well-recognized representatives of insect protein sources. Boukil et al. [116] utilized HHP for the pretreatment of mealworm hydrolysates, demonstrating that a pressure of 380 MPa for 1 min can improve the degree of hydrolysis facilitated by Alcalase. The HHP effect induces protein unfolding, exposing cleavage sites for enzymatic hydrolysis [116]. The consequence of increased hydrolysis includes enhanced solubility and reduced allergenicity of insect proteins [117]. However, Dion-Poulin et al. [117] also reported a negative effect on the degree of hydrolysis. When mealworm and cricket proteins were treated under the same conditions of 380 MPa for 1 min. Different degrees of hydrolysis outcomes were observed. HHP reduced the degree of hydrolysis of mealworm protein hydrolyzed by Alcalase, while cricket protein was unaffected. Furthermore, HHP has been employed in the defatting process to improve the functional properties of insect proteins. Hexane, coupled with a pressure of 500 MPa for 15 min, was used to defat proteins from mealworms and crickets [118]. The significant result demonstrated that HHP enhances the oil-binding capacity of these insect proteins, aligning with the findings reported by Dion-Poulin [117]. Moreover, HHP promotes the total phenolic content of insect proteins when treated with a pressure of 50 MPa for 15 min [118]. HHP increases extractability by disrupting and releasing both hydrophilic and hydrophobic compounds from cells. Limited research has been conducted on the application of HHP to insect proteins. A more extensive body of studies would contribute to a deeper understanding of the mechanisms through which pressure influences the properties of insect proteins. This increased knowledge base is essential for optimizing the application of HHP in the context of insect proteins.

**Table 3:** The effects of HHP on bioactivities, allergenicity, as well as structural, functional, and other properties of alternative protein sources.

Protein Sources	Samples	Extraction Methods	Pressure (MPa)	Time (min)	Effects on Samples			References
					Structural Property	Bioactivities and Allergenicity	Functional and Other Properties	
Algae	*A. platensis* protein extracts	PBS (0.1 M, pH 6.8)	100–600	0–20	-	-	(+) protein solubility (+) C-phycocyanin	[112,113]
		PBS (0.01 M, pH 7)	600	5	-	(−) antioxidant activity	(+) phycobiliproteins	[119]
	*A. platensis* *C. vulgaris*	-	300–600	15	-	-	(+) G′	[114]
	*P. palmata* *S. chordalis* protein extracts	Cellulase (Tris-HCl, pH 5)	400	20	-	(+) antioxidant activity (+) TPC	(+) protein concentration	[115]
	*P. cruentum* protein extracts	Tris-HCl (0.5 M, pH 7)	50–500	5	(−) FI	-	(−) B-phycoerythrin	[120,121]
Insects	Cricket protein extracts	Alkali (pH 10)	500	15	-	-	(+) viscosity	[122]
	Mealworm protein extracts	Protease (0.25%)	380	1	-	(−) allergenicity	(+) DH	[116]
		Ascorbic acid (2%)	70–600	5	(+) particle size (−) FI	-	(+) optical density (+) surface hydrophobicity	[123]
	Cricket and mealworm protein extracts	Alkali (pH 8.5) + Alcalase (3%)	380	1	-	-	(−) DH (−) protein solubility (+) OBC	[117]
		-	500	15	(+) amide II	-	(+) OBC (+) TPC	[118]

Symbols: (−) decrease; (+) increase. Abbreviations: DH (degree of hydrolysis); FI (fluorescence intensity); G′ (storage modulus); OBC (oil-binding capacity); PBS (phosphate buffer saline); TPC (total phenolic content).

## 4. Conclusions and Future Remarks

HHP stands out as an environmentally friendly technology capable of modifying protein functional properties without resorting to thermal methods. These modifications result from structural alterations within proteins induced by HHP. Notably, across various protein sources, the most frequently observed changes include heightened surface hydrophobicity and increased antioxidant activities, often coupled with diminished allergenicity, excluding algal proteins. The enhancement in surface hydrophobicity is attributed to the unfolding of proteins, revealing hydrophobic regions previously concealed within their structures. These alterations significantly impact indicators such as EAI, ESI, and foaming properties. Simultaneously, this phenomenon fosters enzymatic activities, contributing to reduced allergenicity, enhanced bioactivities, and improved digestibility. Consequently, texture profiles encompassing hardness, springiness, chewiness, and gumminess, particularly in proteins from seafood, meat, and poultry products, demonstrate improvements under HHP. However, diverse effects are observed in certain parameters, such as WHC, which is positively influenced in seafood, legumes, and nuts but adversely affected in meat and poultry products. Hence, HHP not only fine-tunes functional properties but also impacts nutritional and allergic attributes. It is noteworthy that the optimal pressure conditions do not consistently correlate with maximal improvement. Appropriate pressure magnitudes offer functional attributes superior to those achieved at maximum pressure in certain samples. Consequently, the optimization of HHP conditions emerges as a key determinant for achieving the utmost functional qualities.

Based on its demonstrated efficacy, HHP should broaden its scope beyond pasteurization, encompassing processes like protein extraction. HHP exhibits the potential to enhance the quality of various protein products, including plant-based proteins, alternative protein sources, and functional foods. Its application enables the production of protein products with preserved nutritional values and bioactivities, coupled with reduced allergenicity. Given HHP’s dual functionality in pasteurization and protein property modification, precise control of optimal HHP conditions becomes imperative to achieve both pasteurization and desired functional properties for each protein product. Additionally, exploring synergistic effects by combining HHP with other innovative techniques, such as ultrasonication and pulsed electric fields, offers opportunities to elevate food quality and extend product shelf-life.

## Figures and Tables

**Table 1:** The effects of HHP on bioactivities, allergenicity, as well as structural, functional, and other properties of plant-based protein sources.

Protein Sources	Samples	Extraction Methods	Pressure (MPa)	Time (min)	Effects on Samples			References
					Structural Property	Bioactivities and Allergenicity	Functional and Other Properties	
Cereals	Buckwheat grains	-	600	30	(+) particle size	(−) antioxidant activity (+) TPC	(−) EAI and ESI (−) foaming capacity and stability (−) G′ (+) WAC and WHC	[18]
	Buckwheat proteins extracts	NaCl (0.086 M)	100–600	1–30	-	(−) IgE binding activity	-	[21]
	Barley β-glucan concentrates	-	300–600	10	(−) particle size	-	(+) G′ (+) WHC and WSI	[22]
	Millet gliadin extracts	NaCl (0.3%)	100–500	10	(−) FI (−) α-helix, random coil (+) β-turn, β-sheets (−) SH	-	(−) protein solubility (−) surface hydrophobicity (−) zeta potential	[23]
	Rice protein extracts	Alkali (pH 10)	100–500	20	(+) FI (−) droplet size	-	(−) creaming index (+) EAI and ESI (+) protein solubility (+) surface hydrophobicity (+) zeta potential	[24]
		PBS (pH 11)	100–300	20	(−) α-helix and β-sheet (+) random coil (+) SH (+) FI	(+) antioxidant activity	(+) EAI and ESI (+) surface hydrophobicity	[19]
		NaOH (0.2 M)	400	15	(+) SH	(+) antioxidant activity	(+) protein content (+) WHC (+) WSI	[20]
	Rice bran protein extracts	Alkali (pH 9)	100–300	30	(−) particle size (+) FI (+) SH	-	(+) EAI and ESI (+) foaming capacity (+) protein solubility (−) zeta potential	[25]
	Quinoa protein extracts	NaCl (0.5 M,	100–600	15	-	-	(+) viscosity (+) G′	[26]
		pH 8)	250–600	15	(−) α-helix, β-sheet (+) β-turn, random coil (−) SH (−) particle size	-	(+) protein solubility (−) surface hydrophobicity	[27]
	Wheat and oat flour	-	150–300	30	-	-	(−) hardness, gumminess, and chewiness (+) L* (+) WHC	[28]
	Wheat gluten proteins	-	100–400	10	(+) SH (+) β-Sheet, random coil (−) α-helix, β-turn	-	(+) G′ (+) gel strength (+) WHC	[29]
			200–500	20	(+) α-helix, β-sheet (−) random coil (−) SH	(−) allergenicity	(+) surface hydrophobicity	[17]
Legumes and nuts	Almond proteins extracts	PBS (pH 7.4)	400–580	3	-	(+) immunoreactivity	(−) protein solubility	[30]
	Cowpea protein extracts	Alkali (pH 10)	200–600	5	(−) DD (−) ∆H (−) FI	-	(+) G′ (+) hardness (−) L* (−) protein solubility (−) viscosity (+) WHC	[31,32,33,34,35]
	Kidney bean protein extracts	Alkali (pH 8)	200–600	15	(−) ∆H	-	(+) EAI and ESI (+) foaming capacity and stability (+) L* (+) G′ (+) WHC	[36]
	Pea protein extracts	Alkali (pH 10)	200–600	5	(−) droplet size (−) FI	-	(+) ESI (−) foaming capacity	[37]
	Pulse protein isolates	-	600	4	(−) DD (−) ∆H	-	(+) ESI (+) foam expansion and stability (+) G’ (−) protein solubility (+) surface hydrophobicity (+) WHC	[38]
	Peanut protein extracts	Alkali (pH 9)	50–200	5	(−) ∆H (+) SH	-	(+) hardness (+) OBC (+) surface hydrophobicity (+) WHC	[39]
		Tris-HCl (50 mM, pH 8.0)	200–600	2.5–20	-	(−) allergenicity	-	[40]
	Walnut protein extracts	Alkali (pH 8.5)	300–600	20	(+) FI (+) SH	-	(+) EAI and (−) ESI (+) foaming capacity and stability (−) protein solubility (+) protein digestibility (+) surface hydrophobicity	[41]
	Soybeans	-	350–550	15	(+) α-helix (−) β-sheet (−) FI	(−) agglutinin activity (−) cytotoxicity	-	[42]
	Soybean protein extracts	Alkali (pH 8)	50–150	12–24 h	-	(+) anti-inflammatory activity	(+) protein yield (+) protein content (+) AA content	[43]
		Alkali (pH 8)	80–300	1–5 h	-	(+) antioxidant activity (+) ACE inhibitory activity	(+) DH (−) surface hydrophobicity	[44]
	Soybean protein isolates	-	200–500	5–20	(−) α-helix, β-sheet (+) random coil (−) FI (+) SH	(−) allergenicity	-	[45]
		-	200–500	15	-	(−) allergenicity	-	[46]
		-	200–400	10	(+) α-helix, random coil (−) β-sheet (−) particle and size (+) SH	-	(+) EAI and ESI (+) protein solubility (+) surface hydrophobicity (−) zeta potential	[47]
		-	250	30	(+) α-helix (−) β-sheet, β-turn (−) SH (+) particle size	-	(+) EAI and (−) ESI (+) foaming capacity and stability (−) hardness (+) protein solubility (+) surface hydrophobicity (+) zeta potential	[48]
		-	200–600	15	(+) α-helix (−) β-sheet (−) SH	-	(+) protein solubility (+) surface hydrophobicity (−) viscosity	[49]
Seeds	Cumin protein extracts	Alkali (pH 9)	200–600	15	(−) FI	-	(−) EAI and ESI (+) surface hydrophobicity (+) protein solubility (+) G’	[50]
	Flaxseed protein extracts	Alkali (pH 10)	600	5–20	(−) FI (+) particle size	-	(+) antioxidant activity	[51]
		Cellulase (1.6 U/mg)	100–300	5–10	(+) FI	-	(+) antioxidant activity	[52]
	Ginkgo seed protein isolates	-	100–700	20	(−) α-helix, β-sheet (+) random coils (+) FI (+) SH	(−) allergenicity	(+) EAI	[53]
	Rapeseed protein extracts	Alkali (pH 11)	400	5–20	-	-	(+) surface hydrophobicity	[54]
Vegetables	Sweet potato protein extracts	NaHSO_3_ (0.1% *w*/*v*)	200–600	15	(−) α-helix, β-turn (+) β-sheet (−) droplet size (−) ∆H	-	(+) surface hydrophobicity (+) EAI and ESI (+) G’ (−) protein solubility (−) creaming stability	[55,56,57,58]
		NaHSO_3_ (10mg/mL)	100–600	20	(+) β-sheet (−) β-turn (−) ∆H	-	-	[59]
		NaHSO_3_ (50 mM)	250–550	15	(−) α-helix (+) β-sheet, random coils (+) SH (−) ∆H	(+) antioxidant activity	(+) surface hydrophobicity	[60]
		NaHSO_3_ (0.1% *w*/*v*)	250–550	30	(+) α-helix (−) β-sheet (−) ∆H (+) SH	-	(+) surface hydrophobicity (+) zeta potential (+) G′ and WHC (+) hardness, springiness, chewiness, and gumminess	[61,62,63,64]
		Protease (6% *w*/*w*)	100–300	60	-	(+) ACE inhibitory activity	(+) DH (+) protein recovery	[65]
		-	300–500	30	-	-	(+) hardness (−) WHC	[66]
		-	300–500	20	-	(+) antioxidant activity	(+) EAI and ESI (+) surface hydrophobicity (+) viscosity	[67]

Symbols: (−) decrease; (+) increase. Abbreviations: AA (amino acid); Angiotensin-converting enzyme (ACE); DD (denaturation degree); DH (degree of hydrolysis); EAI (emulsifying activity index); ESI (emulsifying stability index); FI (fluorescence intensity); G′ (storage modulus), IgE (immunoglobulin E); L* (lightness); NaCl (sodium chloride); NaHSO_3_ (sodium bisulfite); OBC (oil-binding capacity); PBS (phosphate buffer saline); SH (sulfhydryl); TPC (total phenolic content); WAC (water absorption capacity); WHC (water holding capacity); WSI (water solubility index); ∆H (enthalpy change).

## Data Availability

No new data were created or analyzed in this study. Data sharing is not applicable to this article.
